# THE ROLE OF ICT IN ANTIRETROVIRAL THERAPY-RELATED KNOWLEDGE SEEKING AMONG OLDER CHINESE LIVING WITH HIV

**DOI:** 10.1093/geroni/igz038.1539

**Published:** 2019-11-08

**Authors:** Yao Zhang, Xiaoming Li, Shan Qiao, Yuejiao Zhou, Zhiyong Shen

**Affiliations:** 1 Kent State University, School of Information, Kent, Ohio, United States; 2 University of South Carolina, Arnold School of Public Health, Columbia, South Carolina, United States; 3 Guangxi Center for Disease Control and Prevention, Nanning, Guangxi, China (People’s Republic)

## Abstract

Antiretroviral therapy (ART) enables HIV patients to reduce disease progression. ART adherence is closely related to patients’ knowledge about the medical treatment. This study investigated the role of information and communication technologies (ICTs) in supporting ART-related knowledge seeking among older Chinese with HIV, using cross-sectional data collected from 2012 to 2013 in Guangxi, China. Of the 2987 HIV patients, 688 were 45 years or older and going through ART. We used an 11-item scale (α=0.69), which was developed based on existing literature, to assess ART-related knowledge to obtain a composite score (0-11). Less than 5% of the participants sought HIV-related information via computers. Patients with lower ART-related knowledge were more likely to consult medical professionals about the disease via cell phones than those with higher scores (8.28 vs. 9.45, p < 0.001). The results suggest that future interventions should integrate cell phones to promote ART-related knowledge dissemination among older patients.

